# The ABC transporter YejABEF is required for resistance to antimicrobial peptides and the virulence of *Brucella melitensis*

**DOI:** 10.1038/srep31876

**Published:** 2016-08-23

**Authors:** Zhen Wang, Pengfei Bie, Jie Cheng, Lin Lu, Buyun Cui, Qingmin Wu

**Affiliations:** 1 Beijing Key Laboratory of Traditional Chinese Veterinary Medicine, Animal Science and Technology College, Beijing University of Agriculture, Beijing 102206, China; 2 Key Laboratory of Animal Epidemiology and Zoonosis of the Ministry of Agriculture, College of Veterinary Medicine, China Agricultural University, Beijing 100193, China; 3 State Key Laboratory for Infectious Disease Prevention and Control, National Institute for Communicable Disease Control and Prevention, Chinese Center for Disease Control and Prevention, Beijing 102206, China

## Abstract

The ability to resist the killing effects of host antimicrobial peptides (AMPs) plays a vital role in the virulence of pathogens. The *Brucella melitensis* NI genome has a gene cluster that encodes ABC transport. In this study, we constructed *yejA1*, *yejA2*, *yejB*, *yejE, yejF*, and whole *yej* operon deletion mutants, none of which exhibited discernible growth defect in TSB or minimal medium. Unlike their parental strain, the mutants showed a significantly increased sensitivity to acidic stress. The NIΔ*yejE* and NIΔ*yejABEF* mutants were also more sensitive than *B. melitensis* NI to polymyxin B, and the expression of *yej* operon genes was induced by polymyxin B. Moreover, cell and mouse infection assays indicated that NIΔ*yejE* and NIΔ*yejABEF* have restricted invasion and replication abilities inside macrophages and are rapidly cleared from the spleens of infected mice. These findings indicate that the ABC transporter YejABEF is required for the virulence of *Brucella*, suggesting that resistance to host antimicrobials is a key mechanism for *Brucella* to persistently survive *in vivo*. This study provided insights that led us to further investigate the potential correlation of AMP resistance with the mechanisms of immune escape and persistent infection by pathogens.

Multicellular organisms use various defense strategies to protect themselves from microbial infections. Production of antimicrobial peptides (AMPs) is one of these strategies. As an early component of the host response, AMPs modulate the bacterial load and prevent the establishment of an infection^1^. The target of these antimicrobial peptides is postulated to be the cytoplasmic membrane of Gram-positive and Gram-negative bacteria. These peptides insert into the lipid bilayer to generate voltage-gated channels, resulting in the leakage of essential cellular components and, ultimately, the death of the microbe^2^.

The ability of a microbe to prosper within animal host environments requires the capacity to synthesize nutrients not available from host tissues and to avoid or resist being killed by the host niche^3^. *Brucella spp*. are facultative intracellular pathogens that cause abortion and infertility in animals and severe debilitating febrile illness in humans. *Brucella* evolved to exist in host macrophages in the presence of a number of host-imposed stresses, including acidic pH, bactericidal compounds, and low nutrient availability^4^. The ability of brucellae to survive and replicate within macrophages is essential for their virulence^5^, and many stress-associated proteins^6^,^7^,^8^ and virulence determinants^9^ essential for *Brucella* to infect different hosts have been described.

Previous screening of *Brucella* virulence determinants in our lab resulted in the identification of a gene cluster located at chromosome I of the *Brucella melitensis* NI genome that encodes the components of a putative ABC-type microcin C transport system. The operon consists of five genes: *yejA1* (BMNI_I0010) and *yejA2* (BMNI_I0009), which encode putative extracellular solute-binding proteins; *yejB* (BMNI_I0008) and *yejE* (BMNI_I0007), which encode transport system permease components; and *yejF* (BMNI_I0006), which encodes the ABC transporter system ATP-binding protein. Whereas the *Escherichia coli* and *Salmonella Typhimurium* genomes also contain a gene cluster that encodes the components of a putative ABC-type dipeptide/oligopeptide/nickel transport system, this putative operon consists of four genes: *yejA* (b2177 and STM 2216), *yejB* (b2178 and STM 2217), *yejE* (b2179 and STM 2218), and *yejF* (b2180 and STM2219), all of which confer resistance to antimicrobial peptides and contribute to its virulence in *Salmonella*^10^. Bacterial ABC transporter systems are associated with nutrient uptake and the export of toxins and antibiotics, and they also have a potential pathogenic role during host infection^11^. In *Brucella* spp., a polysaccharide ABC transporter is required for *B. abortus* pathogenesis in the murine model^12^. In addition, a predicted ABC transporter, *AbcEDCBA*, of *Brucella ovis* promotes intracellular survival by affecting T4SS protein expression at the post-transcriptional level and, consequently, contributing to *B. ovis* evasion of phagosome/lysosome fusion^13^. It has also been reported that the *yejE* and *yejF* genes of *S. enterica* serovars *Typhimurium* and *Typhi* were up-regulated inside host macrophages^14^,^15^, indicating the importance of these genes inside the host cells. Apart from these studies in *Salmonella*, no studies of the *yejABEF* operon have been performed in *Brucella*, and as a result, not much is known about the function of the *yej* operon in *Brucella*.

The objective of the present study was to investigate the possible functions of *yej* operon genes and determine their role in the virulence of *Brucella*. In this study, we show that mutants of the *yej* operon are more sensitive to acid stress and polymyxin B, have reduced proliferation inside macrophages, and have remarkably decreased virulence in a mouse model.

## Results

### Products of the *yejA1, A2, B, E* and *F* genes in *Brucella* share amino acid sequence similarity with the peptide transporters of *Salmonella*

The *yej* operon genes of *Brucella* are annotated as genes encoding components of a putative ABC transporter system. To examine their potential functions, we searched for the same operon in *Salmonella*. Bioinformatic analysis revealed that the proteins encoded by the *yej* operon share high amino acid sequence identity with those in *Salmonella Typhimurium* (Figure S1). The proteins YejB and YejE of *B. melitensis* NI have relatively high sequence identities, 63.9% and 64.2%, with those in *S. Typhimurium*. Analysis of the *B. melitensis* YejB and YejE sequences revealed the presence of a BPD_transp_1motif that can be defined as a binding-protein-dependent transport system inner membrane component. In addition to containing the BPD_transp_1motif, YejE also contains an OppC_N motif, which was defined as a N-terminal TM domain of oligopeptide transport permease C, similar to that in *Salmonella*. The sequence identity of YejF is 55.4%, and it has the typicalABC_tran (ABC transporter) motif. YejA1 and YejA2 of *B. melitensis* are 35.4% and 34.4% identical to the *Salmonella* YejA. All the YejA proteins of *Brucella* and *Salmonella* contain the SBP_bac_5 motif (Bacterial extracellular solute-binding proteins, family 5 Middle). In addition to the SBP_bac_5 motif, YejA2 also contains a TAT_signal (Twin-arginine translocation pathway signal sequence) motif. The identities of YejABEF among *B. melitensis* NI, *B. melitensis* 16 M, and *B. abortus* 2308 are over 99.5%; the blast results were not described in detail. It was reported that the *yej* operon genes contribute to virulence in *Salmonella* spp^10^,^16^. by counteracting AMPs. Based on these facts, we hypothesized that the transporter system encoded by the *yej* operon might be involved in conferring virulence to *Brucella* and may also be involved in counteracting AMPs, similar to the transporter system encoded by the *yej* operon in *Salmonella*.

### Growth characteristics of *yej* operon gene deletion mutants

**Figure 1:**
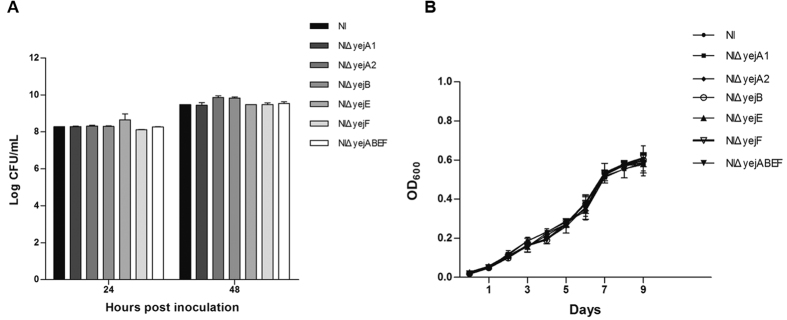
Growth characteristics of the *yej* gene deletion mutants and *B. melitensis* NI in TSB (**A**) and minimal media (**B**). Compared to the wild-type strain, all the *yej* mutants showed similar CFU/mL (TSB) and OD_600_ values (minimal media).

The dynamic growth profiles of the *yej* operon gene deletion mutants and the parent strain NI were determined in TSB and minimal medium. All *yej* mutants grew normally in TSB medium when compared with the parental strain over different time points (Fig. 1A). The minimal medium is a defined medium that contains only carbon and nitrogen nutrients. All these strains were able to grow in the minimal medium, indicating that the inorganic carbon and nitrogen resources provide sufficient nutrients for *Brucella* growth and replication. In addition, the *yej* operon gene deletion mutants showed similar growth profiles in minimal medium (Fig. 1B, *P* > 0.05), indicating that the *yej* operon gene deletion mutants were not compromised in their capacities to utilize limited nutrition.

### YejABEF proteins contribute to resistance to acidic conditions

**Figure 2:**
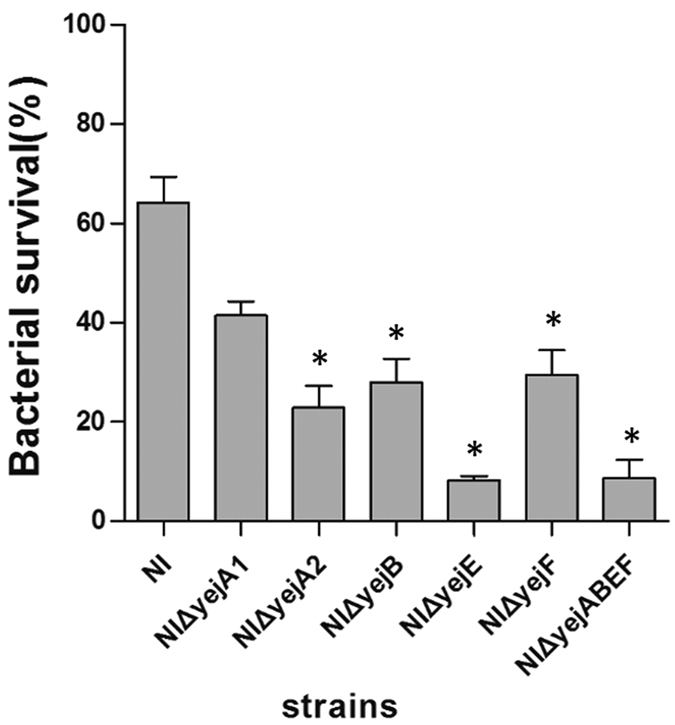
Survival rates of *yej* gene deletion mutants and *B. melitensis* NI under acidic conditions. After 2 h of exposure to pH 3.4 minimal media, the survival rates of each strain were calculated. The presented values represent the means of three experiments performed in duplicate, and the error bars indicate the SD. Significant differences between the strains are indicated by an asterisk (*p* < 0.05).

To investigate the effect of the *yej* operon on acid stress tolerance, brucellae survival was evaluated under a reduced pH level. After a 2-h exposure to a pH of 3.4 at 37 °C, all *yej* operon gene deletion mutants and the parental strain showed reduced bacterial viability. *B. melitensis* NI showed a 36% decrease in bacterial viability, whereas the *yej* operon gene deletion mutants NIΔ*yejA1*, NIΔ*yejA2*, NIΔ*yejB*, NIΔ*yejE*, NIΔ*yejF*, and NIΔ*yejABEF* showed decreases of 58.6%, 77.2%, 72%, 91.7%, 70.5%, and 91.3%, respectively. These results showed that except for NIΔ*yejA1*, all other *yej* operon gene deletion mutants were more sensitive to acidic conditions than the parental NI strain (Fig. 2, *P* < 0.05).

### Deletion of the *yejE* gene confers susceptibility to polymyxin B

**Figure 3:**
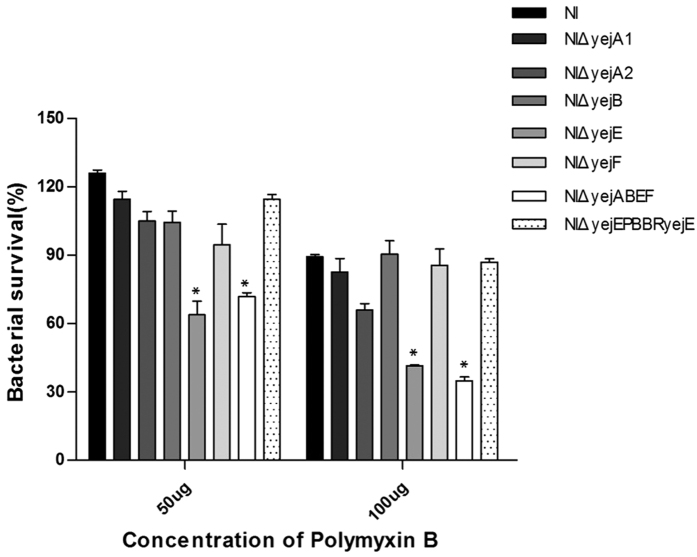
Sensitivity of *yej* gene deletion mutants and *B. melitensis* NI to different concentrations of polymyxin B. The data are representative of three independent experiments. Significant differences between every mutant and parental strain are indicated by an asterisk (*P* < 0.05).

Based on our hypothesis that the *yej* operon may confer resistance to AMP, we investigated the sensitivity of each *yej* gene deletion mutant to polymyxin B. Polymyxin B is a cationic peptide derived from *Paenibacillus polymyxa* that interacts with the outer and inner membranes of Gram-negative bacteria in a similar fashion to many AMPs^17^,^18^. The whole *yej* operon deletion mutant NIΔ*yejABEF* was more sensitive to both concentrations of polymyxin B when compared with the parental NI strain (Fig. 3, *P* < 0.05), confirming the role of the *yej* operon in conferring the resistance of *Brucella* to AMP. The NIΔ*yejE* mutant also showed an increased sensitivity to polymyxin B (Fig. 3, *P* < 0.05). Surprisingly, the mutants NIΔ*yejA*1, NIΔ*yejA*2, NIΔ*yejB*, and NIΔ*yejF* did not show any increased sensitivity when compared with the parental NI strain in survival upon treatment with polymyxin B at both final concentrations (Fig. 3, *P* > 0.05). Introducing pBBR*yejE* into the mutant NIΔ*yejE* restored the polymyxin B resistance to the level of the parental strain, suggesting that the transport system permease YejE played a key role in the AMP resistance of *B. melitensis*, while the proteins YejA1, YejA2, YejB, and YejF are dispensable in this aspect.

**Figure 4:**
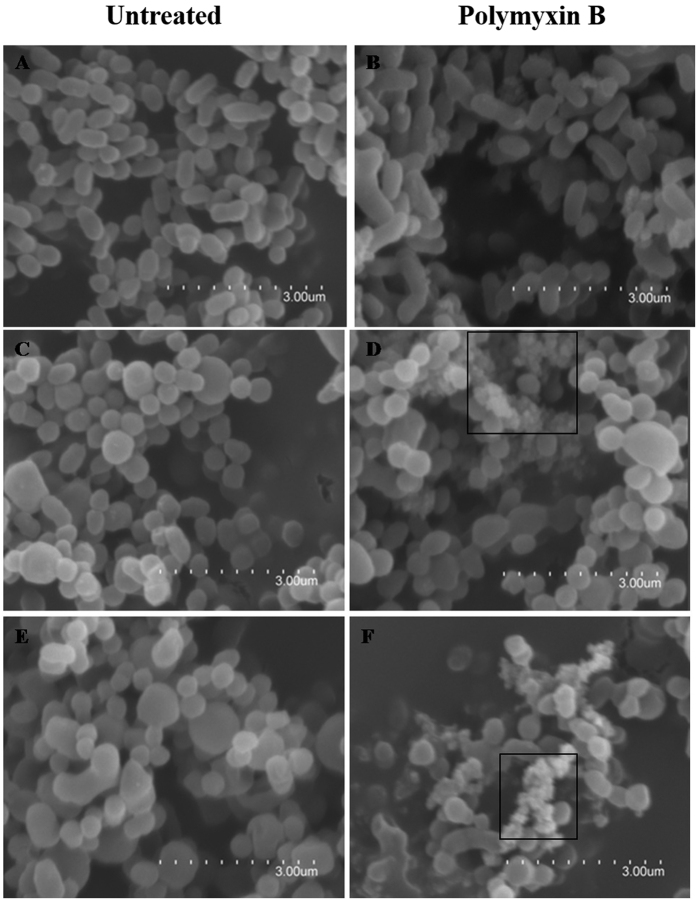
Scanning electron microscopic images of strains *B. melitensis* NI (**A,B**), NIΔ*yejE* (**C,D**), and NIΔ*yejABEF* (**E,F**) treated with polymyxin B. The box indicates disrupted bacteria. Scale bar, 3 μm.

Exposure of bacteria to high concentrations of cationic peptides in most cases results in membrane damage and bacterial death. To confirm the NIΔ*yejE* and NIΔ*yejABEF* mutants’ hypersensitivity to cationic peptides, we evaluated the morphology of bacteria treated with polymyxin B using a scanning electron microscope. In the case of the mutants NIΔ*yejE* and NIΔ*yejABEF*, we could see many damaged bacteria with membrane irregularities (box, Fig. 4), and many bacteria formed an irregular mass of debris with their extruded cytoplasm.

### *Yej* operon genes are induced by Polymyxin B

**Figure 5:**
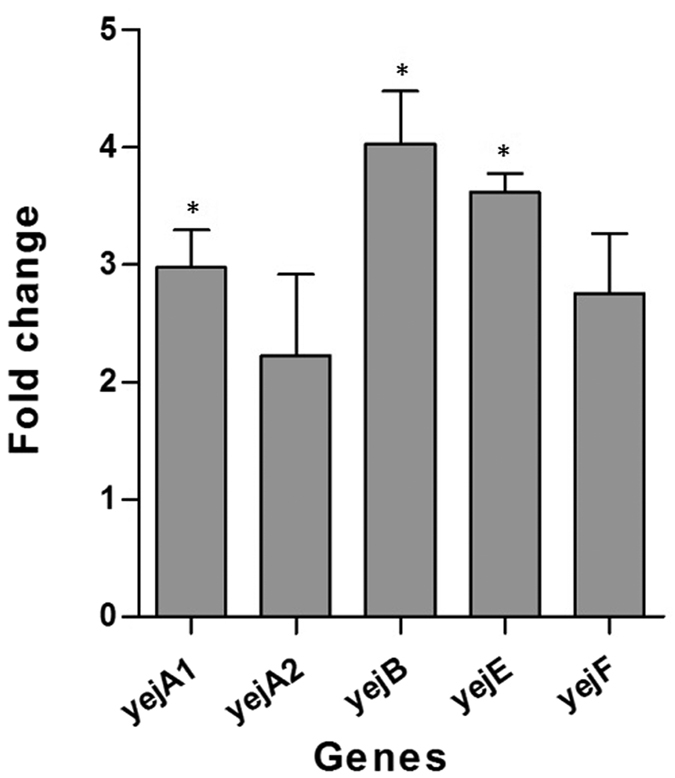
Real-time PCR analysis of each *yej* operon gene expression level in *B. melitensis* NI under polymyxin B treatment. 16S rRNA was used as a reference gene to normalize the expression levels of the target gene. The fold change is expressed as a ratio of normalized gene expression levels under polymyxin B treatment to those under normal culture conditions. Significant differences between the gene expression levels are indicated by an asterisk (*P* < 0.05).

The polymyxin B sensitivity assay confirmed the role of the *yej* operon in conferring AMP resistance in *Brucella*. It is likely that *Brucella* encounters antimicrobial peptides within host micro-environments during *in vivo* infection. These peptides may contribute to environmental signals that trigger changes in bacterial gene expression. We therefore examined the cationic peptide polymyxin B to determine whether it could stimulate *yej* operon gene expression. A summary of the relative *yej* operon gene expression levels observed in different samples is presented in Fig. 5. As shown in Fig. 5, we observed that the expression of *yejA1*, *yejA2*, *yejB*, *yejE*, and *yejF* in *B. melitensis* NI was induced by polymyxin B and that the expression of *yejA1*, *yejB*, and *yejE* was significantly higher in samples treated with Polymyxin B than in samples without peptide (Fig. 5, *P* < 0.05). The *yejA1*, *yejB*, and *yejE* expression levels increased by approximately 3.0, 4.0, and 3.6-fold, respectively, under polymyxin B inducement compared to the untreated control.

### YejE is required for the replication of *B. melitensis* NI in J774.A1 macrophages

**Figure 6:**
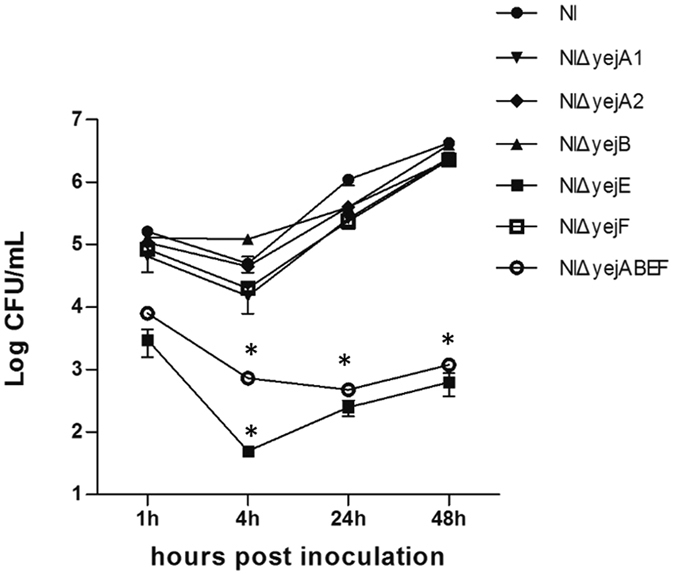
Multiplication of *yej* gene deletion mutants and *B. melitensis* NI in J774.A1 macrophages. At the indicated hour p.i., the number of intracellular bacteria was measured and expressed as log_10_ CFU/mL. The presented values represent the means of three experiments performed in duplicate, and the error bars indicate the SD. Significant differences between every mutant and parental strain are indicated by an asterisk (*P* < 0.05).

The role of the *yej* operon in *in vitro* sensitivity to the acid and AMP levels that are predicted to be encountered in host macrophages prompted us to investigate the effect of the YejABEF proteins on the virulence of *B. melitensis*. First, we assessed the intracellular survival of the *yej* operon gene deletion mutants. As shown in Fig. 6, at 1 h post-infection, the macrophages infected with the NIΔ*yejE* and NIΔ*yejABEF* mutants showed lower bacterial loads than the macrophages infected with *B. melitensis* NI, NIΔ*yejA*1, NIΔ*yejA*2, NIΔ*yejB*, and NIΔ*yejF* (*P* < 0.05), which indicated that there were significant variations in the ability of NIΔ*yejE* and NIΔ*yejABEF* to invade macrophages. At 4 h post-infection, the intracellular bacterial loads of each strain decreased to a different degree. The mutant NIΔ*yejE* showed an especially sharp reduction in intracellular bacterial number. However, after 4 h post-infection, the CFUs of *B. melitensis* NI, NIΔ*yejA*1, NIΔ*yejA*2, NIΔ*yejB*, and NIΔ*yejF* increased rapidly, and the CFUs of NIΔ*yejE* also had a trend toward recovery, while the numbers of intracellular bacterial CFUs in NIΔ*yejABEF*-infected cells continuously decreased. At the end of the test, the number of recovered NIΔ*yejE* and NIΔ*yejABEF* was four orders of magnitude lower than that recovered with *B. melitensis* NI, NIΔ*yejA*1, NIΔ*yejA*2, NIΔ*yejB*, and NIΔ*yejF*. These data showed that the NIΔ*yejE* and NIΔ*yejABEF* mutants have restricted invasion and replication abilities inside J774.A1 macrophages, and therefore they exhibited reduced virulence *in vitro*. However, the NIΔ*yejA*1, NIΔ*yejA*2, NIΔ*yejB*, and NIΔ*yejF* mutants did not show any such defect, which is in agreement with the results of the polymyxin B sensitivity experiments. Thus, it was clear that the *yej* operon confers the ability of *B. melitensis* to proliferate inside macrophages, and more specifically, that the protein YejE played a key role in this aspect.

### YejE contributes to the virulence of *B. melitensis* NI in mice

**Figure 7:**
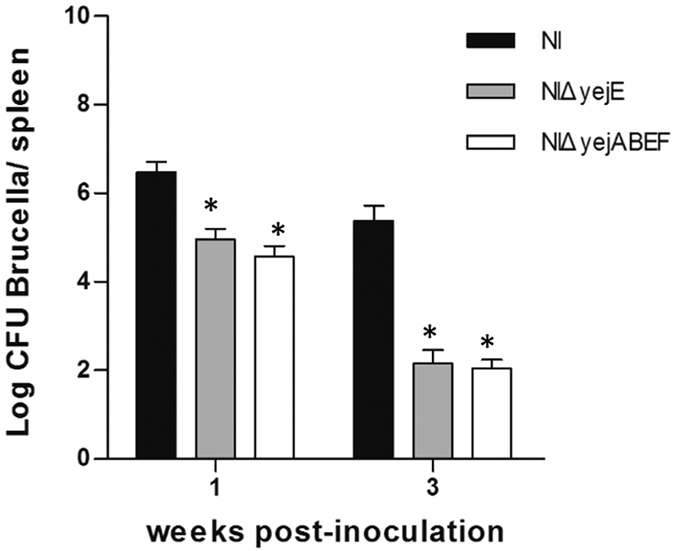
Survival of *yej* gene deletion mutants and *B. melitensis* NI in mice. Ten mice were inoculated with each strain at a dose of 10^6^ CFU/mouse. Five mice/group were euthanized at 1 and 3 weeks post-inoculation, and the virulence of each strain was determined based on the number of CFUs recovered from the spleen, which was expressed as the mean ± SD (n = 5) of individual log_10_ CFU/spleen values. Significant differences between every mutant and parental strain are indicated by an asterisk (*P* < 0.05).

Having demonstrated that yejE is necessary for the replication of *Brucella* in macrophages, we next investigated the virulence of the mutants NIΔ*yejE* and NIΔ*yejABEF* in a murine model. As shown in Fig. 7, we observed a large reduction (above a 2-log difference) in the spleen bacterial load at 1 week post-inoculation in mice inoculated by NIΔ*yejE* and NIΔ*yejABEF* compared to mice infected with *B. melitensis* NI. At 3 weeks post-infection, sharp reductions of the spleen bacterial load were observed in mice infected with NIΔ*yejE* and NIΔ*yejABEF*, whereas more than 10^5^ CFUs of *Brucella* remained in the spleens of mice infected with *B. melitensis* NI. These results indicated that the mutants NIΔ*yejE* and NIΔ*yejABEF* were avirulent in mice, which is consistent with the results of the macrophage infection assay.

## Discussion

As an essential aspect of the host’s innate immune defenses, antimicrobial peptides are largely produced by macrophages, neutrophils, and mucosal epithelial cells^19^, which are at the front line of host defense and play critical roles both in reducing the microbial load early during infection and in linking innate to adaptive immunity. Thus, bacteria have evolved different strategies to resist AMPs, such as remodeling the bacterial outer membrane surface, exporting AMPs via multiple transferable resistance-mediated efflux pumps, secreting exoproteases for AMP degradation, and releasing proteins to adsorb extracellular AMPs^1^. The ability of pathogenic bacteria to resist being killed by antimicrobial peptides in different host niches may therefore contribute to their virulence^1^. Successful pathogens, including intracellular pathogens, have evolved different mechanisms to evade the microbicidal effects of these molecules. For example, the facultative intracellular pathogen *S. typhimurium* harbors several proteins that enable it to resist being killed by peptides, and strains with mutations in the corresponding genes are avirulent^16^. Two putative ATP-binding cassette (ABC) transporters encoded by the *sapABCDF* operon and *yejABEF* are required to counteract AMPs, contributing to the virulence of *Salmonella*^10^,^16^. In addition, it was also reported that the Sap transporter equips *Haemophilus* to resist AMPs by mediating the import and subsequent degradation of antimicrobial peptides^20^. Because the host produces AMPs to control bacterial growth, leading to bacterial clearance, and bacterial AMP resistance mechanisms provide advantages to pathogens, leading to disease progression. The outcome of bacterial infection is determined by the balance between bacterial resistance mechanisms and host defense responses during infection.

In this study, the *yej* operon genes (*yejA1*, *yejA2*, *yejB*, *yejE*, and *yejF*) in the *Brucella* genome are annotated as gene-encoding components of a putative ABC-type microcin C transport system. To better understand the role of *yej* genes in *Brucella* in resistance to being killed by antimicrobial peptides, we first characterized the regulation of *yej* genes in *B. melitensis* NI under polymyxin B treatment. The *yejA1*, *yejA2*, *yejB*, *yejE*, and *yejF* expression levels, as measured by RT-PCR, were all increased in a medium containing polymyxin B. The up-regulation of *yej* genes by polymyxin B demonstrated the direct response of *Brucella* to the presence of antimicrobial peptides, which was similar to *mig*-*14*, a *Salmonella* gene strongly induced by polymyxin B^21^, suggesting that the expression of *yej* genes in *B. melitensis* was directly induced by polymyxin B.

We further demonstrate that the *yej* operon of *Brucella* also confers resistance to polymyxin B. It was observed that both the whole *yej* operon deletion mutant NIΔ*yejABEF* and the *yejE* deletion mutant NIΔ*yejE* were more sensitive to polymyxin B (Fig. 3), revealing significantly lower survival rates compared to the parental strain NI. This observation is similar to the results of Eswarappa *et al*., who observed that *S. Typhimurium yej* mutants were sensitive to polymyxin B^10^. In agreement with the results of the polymyxin B sensitivity experiment, the NIΔ*yejABEF* and NIΔ*yejE* mutants showed restricted invasion and replication abilities inside J774.A1 macrophages, but the NIΔ*yejA*1, NIΔ*yejA*2, NIΔ*yejB*, and NIΔ*yejF* mutants did not exhibit reduced virulence *in vitro*.

The different phenotypes of the NIΔ*yejA*1, NIΔ*yejA*2, NIΔ*yejB*, and NIΔ*yejF* mutants were unexpected when compared with NIΔ*yejE* and NIΔ*yejABEF*, as periplasmic-binding proteins are essential for the function of bacterial importers. However, it has been reported that some histidine and maltose transporter mutants can also function independent of their periplasmic-binding proteins^22^. Thus, it is possible that YejA, YejB and YejF are dispensable for the function of the Yej transporter, at least in counteracting AMP and intracellular survival. Similar to the *yej* mutants of S. *Typhimurium*, the *yej* mutants of *Brucella* also did not show any discernible growth defect in TSB or minimal media.

Within host macrophages, *Brucella* replicate in BCVs associated with the endoplasmic reticulum. While early acidification of vacuolar compartments has been shown to be essential for the intracellular survival of *Brucella*^23^, the pH of phagocytic vacuoles has also been observed to decrease rapidly to 4.0–4.5^24^, which indicates that *Brucella* must adapt to the low-pH environment. Therefore, we determined the acid resistance of *yej* mutants. The results indicated that *yej* mutants were more sensitive to acidic conditions than the parental strain.

The results indicated that the *yej* mutants were both sensitive to acidic stress and AMPs, but the linkage between these two phenomena has received little attention thus far. Preliminary work confirmed that *asp24* was related to acid shock, and the optimal expression levels of Asp24 were reached at pH values below 4.0, which indicated an active role for this protein in resistance to acidic environments^25^. In addition, a previous study in our lab also confirmed that a *cspA* mutant was sensitive to acidic stress^26^, and a *manBA* mutant was sensitive to polymyxin B (data not published). To explain the connection between acidic stress and AMP tolerance, the mutants with the respective deletions of the *asp24*, *manBA*, and *cspA* genes previously constructed by our lab, were compared with NIΔ*yejE* and NIΔ*yejABEF* to evaluate their resistance to acidic and AMP stresses. The results indicated that the *manBA* mutant, NIΔ*yejE* and NIΔ*yejABEF* were all sensitive to acidic stress and polymyxin B, while the *asp24* and *cspA* mutants were merely sensitive to acidic environments (data not shown). Thus, we inferred that the strains that were sensitive to AMPs were the most likely to be sensitive to acidic stress, while the strains that were sensitive to acidic stress were not always sensitive to polymyxin B. Notably, the mutants might also have decreased resistance to acidic stress if they were susceptible to AMPs.

To further evaluate the virulence of the *yej* mutants, we tested the *in vivo* survival of NIΔ*yejE* and NIΔ*yejABEF* in a mouse model. After 3 weeks of infection, there was a significant decrease in the bacterial burden in the spleens of mice infected with the NIΔ*yejE* and NIΔ*yejABEF* mutants compared to mice infected with the parental strain. This observation clearly indicates that the transporter encoded by the *yej* operon is important for *in vivo* infections and persistent survival. We inferred that the inability of the NIΔ*yejE* and NIΔ*yejABEF* mutants to replicate within host macrophages and the attenuation of their growth in mice might be due to the sensitivity of these strains to AMPs and acid stresses that would presumably be encountered by bacteria during infection. Because certain AMPs are capable of mediating changes in the gene expression profiles of bacterial virulence factors, they might influence bacterial tolerance of harsh conditions in the host. Thus, these results suggested that resistance to host defense responses is important not only in initiating infection but also in maintaining persistent infection.

As a first line of innate defense, AMPs serve to limit bacterial colonization during infection. However, bacteria, especially intracellular pathogens, have evolved different strategies to sense and resist the functions of AMPs, as in the novel finding that the *yej* operon is important for resistance to AMPs and the persistent survival of *Brucella melitensis*. This study demonstrated that resistance to host antimicrobials is a key mechanism of persistent infection for *Brucella*, and it might be an important way for pathogens to evade the host defense system and persistently survive *in vivo*. This novel finding also led us to further investigate the potential correlation of antimicrobial peptide resistance with the mechanisms of immune escape and persistent infection by pathogens.

## Methods

### Ethics statement

All animal research was approved by the Beijing Association for Science and Technology. The approval ID is SYXK (Beijing) 2007–0001, and the animal research complied with the Beijing Laboratory Animal Welfare and Ethics guidelines of the Beijing Administration Committee of Laboratory Animals.

### Bacterial strains and media

All bacterial strains and plasmids used in this study are listed in Table 1. *Brucella* strains, including the parental strain and the derived mutants, were routinely grown or incubated in TSB, tryptic soy agar (TSA) or a minimal medium that has been described previously^26^. The pH levels of the minimal media were adjusted with HCl. *Escherichia coli* strains were grown on Luria-Bertani (LB) plates overnight at 37 °C with or without supplemental ampicillin (100 mg/liter) and chloromycetin (30 mg/liter). All work with live *Brucella* strains was performed in the biosafety level 3 facilities of the Chinese Center for Disease Control and Prevention.

### Mice

Female BALB/c mice (aged 4 to 6 weeks) were purchased from the Weitong Lihua Laboratory Animal Services Centre (Beijing, China), bred in individually ventilated cage rack systems, and subsequently transferred to the biosafety level 3 facilities of the Chinese Center for Disease Control and Prevention at the beginning of the experiments. All experiments involving animals followed the regulations of the Beijing Administration Office for Laboratory Animals.

### Construction of *yej* operon gene deletion mutants

Gene knockout constructs were used as described previously^26^. This method has been used successfully to knock out single genes as well as an entire operon. The primers used for each gene deletion are shown in Table S1. The gene deletion mutants were verified by PCR and sequencing analysis and are hereafter referred to as NIΔ*yejA1*, NIΔ*yejA2*, NIΔ*yejB*, NIΔ*yejE*, NIΔ*yejF*, and NIΔ*yejABEF*.

### Construction of the complemented *yejE* deletion mutant strain

To construct the complemented strain of NIΔ*yejE*, the complete *yejE* gene and the promoter sequences were amplified from *B. melitensis* NI genomic DNA via PCR using the primers shown in Table S1. The resulting PCR products were digested with *Sma*I and *Pst*I and then ligated into the pBBR1MCS plasmid digested with the same enzymes. The resulting recombinant vector, pBBR*yejE*, was subsequently electroporated into NIΔ*yejE* to complement the function of YejE. The complementation strains, loaded with pBBR*yejE*, were selected on TSA plates containing chloromycetin. Finally, the selected complementation strains were verified through PCR and designated as NIΔ*yejE*PBBR*yejE*.

### *In vitro* growth characteristics in TSB and minimal medium

To monitor extracellular growth, one colony from each strain (the parental strain and all gene deletion mutants) was inoculated into 5 mL of TSB medium and grown to mid-log phase in a shaking incubator at 37 °C. The cultures were then adjusted to the same concentration (CFU/mL) and subsequently used for growth curve analysis. A 20-μL sample of each strain was inoculated into 5 mL of minimal medium or TSB, followed by incubation at 37 °C. Growth was monitored based on the CFU/mL and OD_600_ values. Growth characteristics were evaluated by analyzing the growth of each strain at different time points.

### Acid resistance experiment

The acid challenge assay was performed as previously described with some modifications^27^. Exponentially growing bacteria of each strain were adjusted to 10^9^ CFU/mL, from which a 200-μl sample of each strain was concentrated, washed, and then resuspended in 200 μl of pH 4.4 minimal medium (for submitted cultures) and incubated for 2 h at 37 °C. Next, the submitted cultures were washed and resuspended in pH 3.4 minimal medium for challenge and incubated for 2 h at 37 °C. The cultures were then immediately serially diluted and plated to determine their viability post-challenge. The survival percentage was calculated by dividing the CFUs obtained 2 h post-acid challenge by those obtained prior to the acid challenge and multiplied by 100.

### Polymyxin B sensitivity assay

Polymyxin B (Sigma, USA) sensitivity assays were performed in triplicate essentially as described with some modification^28^. Essentially, exponentially growing bacteria of each strain were adjusted to 10^4^ CFU/mL, from which a 200-μL sample of each strain was concentrated, washed, and resuspended in 200 μL minimal medium and then mixed with 200 μL of different concentrations of polymyxin B (the final concentrations in the wells were 50 and 100 ug/mL). After 1 h of incubation at 37 °C, the percent survival was calculated as the initial input bacterial CFUs relative to bacterial CFUs after challenge.

### *yej* gene expression levels in *B. melitensis* NI under polymyxin B treatment

The expression levels of the *yej* operon genes in *B. melitensis* NI under AMP induction were detected via RT-PCR. *B. melitensis* NI was grown to the exponential phase in 20 mL of TSB at 37 °C in a shaking incubator. Ten milliliters of the sample bacterial were treated with polymyxin B (100 ug/mL) for 1 h at 37 °C, and another 10 mL of the sample was maintained at 37 °C for 1 h. Then, total RNA samples were prepared for the treated and untreated *B. melitensis* strains. DNA was removed using DNase (Ambion, Foster City, CA), and the RNA samples were reverse transcribed into cDNA using random oligonucleotide hexamers and the Fermentas First-Strand cDNA synthesis kit (Thermo Fisher Scientific, Bremen, Germany) according to the manufacturer’s protocol. The resulting cDNA samples were subjected to quantitative real-time PCR using the Power SYBR Green PCR System. The sense and anti-sense primers for each *yej* operon gene are shown in Table S1. The expression level of the 16S rRNA gene was used to normalize all of the obtained values.

### Scanning electron microscopy

Each strain, including *B. melitensis* NI, NIΔ*yejE*, and NIΔ*yejABEF* (approximately 10^8^ CFU), was treated with 100 ug/mL polymyxin B at 37 °C for 1 h and fixed using 4% glutaraldehyde in 0.1 M phosphate buffer at 4 °C for 4 h or overnight. Then, the samples were fixed using 2% glutaraldehyde in a 0.1 M phosphate buffer at 4 °C for 1 h. The samples were subsequently washed and dehydrated in a series of ethanol washes (70% for 30 min, 90% for 30 min, and 100% for 30 min twice) and air-dried prior to sputter coating with gold. The samples were then analyzed using a scanning electron microscope^29^.

### Cell infection assay

To investigate the intracellular survival of the parental strain and each *yej* gene mutant, J774.A1 murine macrophage infection assays were performed as previously reported with some modifications^26^. Briefly explained, monolayers of cells were cultured in 24-well plates and infected with each strain at a multiplicity of infection (MOI) of 200 CFU. To synchronize the infection, the infected plates were centrifuged at 1,000 rpm for 5 min at room temperature, followed by a 20-min incubation at 37 °C in an atmosphere containing 5% (vol/vol) CO_2_. Then, the cells were washed three times with PBS and incubated in a medium containing gentamycin (50 μg/ml) at 37 °C under 5% CO_2_ until the end of the infection period. At 1 h, 4 h, 24 h, and 48 h p.i., the cells were washed and lysed in sterile 0.5% (vol/vol) Tween 20 water. The number of surviving intracellular bacteria was then determined through serial dilution, followed by plating on TSA or TSA supplemented with chloromycetin. Three replicate wells for each strain were evaluated at each time point. The results presented in this paper represent the averages from at least three separate experiments.

### Virulence in BALB/c mice

Ten mice were intraperitoneally inoculated with a dose of 10^6^ CFU of NIΔ*yejE*, NIΔ*yejABEF*, and *B. melitensis* NI in 0.1 ml of phosphate-buffered saline (PBS). Five infected mice from each infected group were randomly selected and euthanized via carbon dioxide asphyxiation at 1 and 3 weeks post-inoculation. At each time point, the spleens were collected aseptically, homogenized in 1 ml of PBS, and then serially diluted to isolate the bacteria. The bacteria recovered from the spleens were enumerated to evaluate the survival of each strain in mice. The results are presented as the mean number of CFU per spleen±the standard deviation (SD) in each group. If no bacteria grew in the undiluted homogenized sample, the spleen was assumed to contain less than 5 bacteria, i.e., falling below the limit of detection of 5 CFU/spleen.

### Statistical analysis

Student’s *t*-test was performed to analyze the data from the mouse virulence experiments, and ANOVA was performed for the growth curve analysis, cellular infections, and acid and polymyxin B sensitivity response assays. A *P* value of <0.05 was considered to represent a significant difference.

## Additional Information

**How to cite this article**: Wang, Z. *et al*. The ABC transporter YejABEF is required for resistance to antimicrobial peptides and the virulence of *Brucella melitensis*. *Sci. Rep*. **6**, 31876; doi: 10.1038/srep31876 (2016).

## Supplementary Material

Supplementary Information

## Figures and Tables

**Table 1:** Bacterial strains and plasmids.

Strain or Plasmid	Characteristic(s)	Source or Reference
***Brucella***		
*B. melitensis* NI	Epidemic strain, smooth	This lab
NIΔ*yejA1*	*yejA1*-deletion mutant of NI	This study
NIΔ*yejA2*	*yejA2*-deletion mutant of NI	This study
NIΔ*yejB*	*yejB*-deletion mutant of NI	This study
NIΔ*yejE*	*yejE*-deletion mutant of NI	This study
NIΔ*yejF*	*yejF*-deletion mutant of NI	This study
NIΔ*yejABEF*	*yej* operon-deletion mutant of NI	This study
NIΔ*yejE*PBBR*yejE*	Complementation strain of NIΔ*yejE*	This study
***E. coli***		
DH10B	F- *mcr*A Δ(mrr-hsdRMS-mcrBC) Φ80dlacZΔM15 ΔlacX74 endA1 recA1 deoR Δ(ara, leu)7697 araD139 *gal*U *gal*K *nup*G *rps*L(Strr) *nup*G	Invitrogen
**Plasmids**		
pEX18AP	*sac*B, *bla*, Amp^r^	
pBBR1MCS	Broad-host-range plasmid, Cm^r^	
pEX18ApΔ*yejABEF*	*yej* operon gene deleting vector	This study
pBBR*yejE*	*yejE* gene complementing vector	This study
