# Supplementary material for: The ABC transporter YejABEF is required for resistance to antimicrobial peptides and the virulence of Brucella melitensis
Source: Sci Rep. 2016 Aug 23;6:31876. doi: 10.1038/srep31876 (PMC4994006; doi:10.1038/srep31876)
Supplement: Supplementary Information [file srep31876-s1.pdf]

# The ABC transporter YejABEF is required for resistance to antimicrobial peptides and the virulence of *Brucella melitensis*

Zhen Wang[1], Pengfei Bie[2], Jie Cheng[1], Lin Lu[1], Buyun Cui[3], Qingmin Wu[2]*

[1]*Beijing Key Laboratory of Traditional Chinese Veterinary Medicine, Animal Science and Technology College, Beijing University of Agriculture, Beijing 102206, China*

[2]*Key Laboratory of Animal Epidemiology and Zoonosis of the Ministry of Agriculture, College of Veterinary Medicine, China Agricultural University, Beijing 100193, China*

[3]*State Key Laboratory for Infectious Disease Prevention and Control, Collaborative Innovation Center for Diagnosis and Treatment of Infectious Diseases, National Institute for Communicable Disease Control and Prevention, Chinese Center for Disease Control and Prevention, Beijing102206, China*

*Corresponding authors. Tel.: +86-10- 6273-3901

*E-mail address:* wuqm@cau.edu.cn(Q.M. Wu)

**Supplemental Materials**

**Supplemental Table S1** Primers used in this study.

| Primer Name | Sequence (restriction enzyme used)（5'-3'） | Locus (gene) |
|---|---|---|
| **Primers used for RT-PCR** | | |
| pWU 855F | TACCAGCCCTTGACATCC | 16S rRNA |
| pWU 856R | TCATCCCCACCTTCCTCT | |
| RT-061 | AGAAGGCGAAACCGTAGC | BMNI_I0006 |
| RT-062 | GTGAAGCGGATTGAGCGAT | |
| RT-071 | GTTACTGGTCGCTGTGGATT | BMNI_I0007 |
| RT-072 | TCGTTATTGACGGTGTTGT | |
| RT-081 | ATTCCACTCGGCATTCG | BMNI_I0008 |
| RT-082 | GAAATCGGGCGAGGTG | |
| RT-091 | CGGTGCTTACAAGGTCG | BMNI_I0009 |
| RT-092 | CGATGCTGCCTTTCTTG | |
| RT-101 | GTGGCGGGATGCTTTA | BMNI_I0010 |
| RT-102 | GCACATTGAACGACCAGA | |
| **Primers used for gene deletion** | | |
| WD0285 | AACTGCAGCCCCGCCGTGATAGGTCTT | BMNI_I0006 |
| WD0286 | GCGGATCGGCGATACGAAGGGAAATA | |
| WD0287 | CGATCCGCTACTCATTG | |
| WD0288 | CG GGATCC TGGTCGTCAATCCCAG | |
| WD0289 | AACTGCAG AGCAGTTTGGGCTTGTTG | BMNI_I0007 |
| WD0290 | GACGTGTGATGGGTCAATGAGTAGC | |
| WD0291 | CACACGTCCCTCCGGTC | |
| WD0292 | CG GGATCC GGCGGTTCCTTCTTTGAC | |
| WD0297 | AACTGCAG AATGCGGAAGCCATAGAG | BMNI_I0008 |
| WD0298 | TCCAGCTGTGACCGACACCGCACTC | |
| WD0299 | CAGCTGGACCTTATCCG | |
| WD0300 | CG GGATCC TGCGAGCCAGTATCAG | |
| WD0293 | AACTGCAG CTTGCGAATGCCGAGTG | BMNI_I0009 |
| WD0294 | TCCTTTGCTTACCGGAAAGTGGCGA | |
| WD0295 | GCAAAGGAATGTCATTC | |
| WD0296 | CG GGATCC GGCAATCCGTTCACCA | |
| WD0301 | AACTGCAG GGCAGACGATTACAAAG | BMNI_I0010 |
| WD0302 | TTCAGGCCCCAGTTTCTCCACGTTTTG | |
| WD0303 | GGCCTGAACGAGAATG | |
| WD0304 | CG GGATCC AGGTGAATGCCACATCG | |

| | | |
|---|---|---|
| WD0097 | GGGGTACC GCGATACGAAGGGAA | BMNI_I0006-10 |
| WD0098 | CGAAGGTGCCGCCGTGATAGGTCTT | |
| WD0099 | CACCTTCGGTTTTGCTCCTG | |
| WD0100 | CG GGATCC GGGATCGAACCTGAAGAC | |
| **Primers used for gene complementation** | | |
| C0007-1 | CGGGATCCATGACCGACACCGCACTC | BMNI_I0007 |
| C0007-2 | AACTGCAGTCATAAAAATGCCTTTCGC | |
| C0007-P1 | TCCCCCGGGCTTTACGGCTGGAATTTTC | BMNI_I0007 promoter |
| C0007-P2 | CGGGATCCTCTCCACGTTTTGTTCCAG | |

A

B

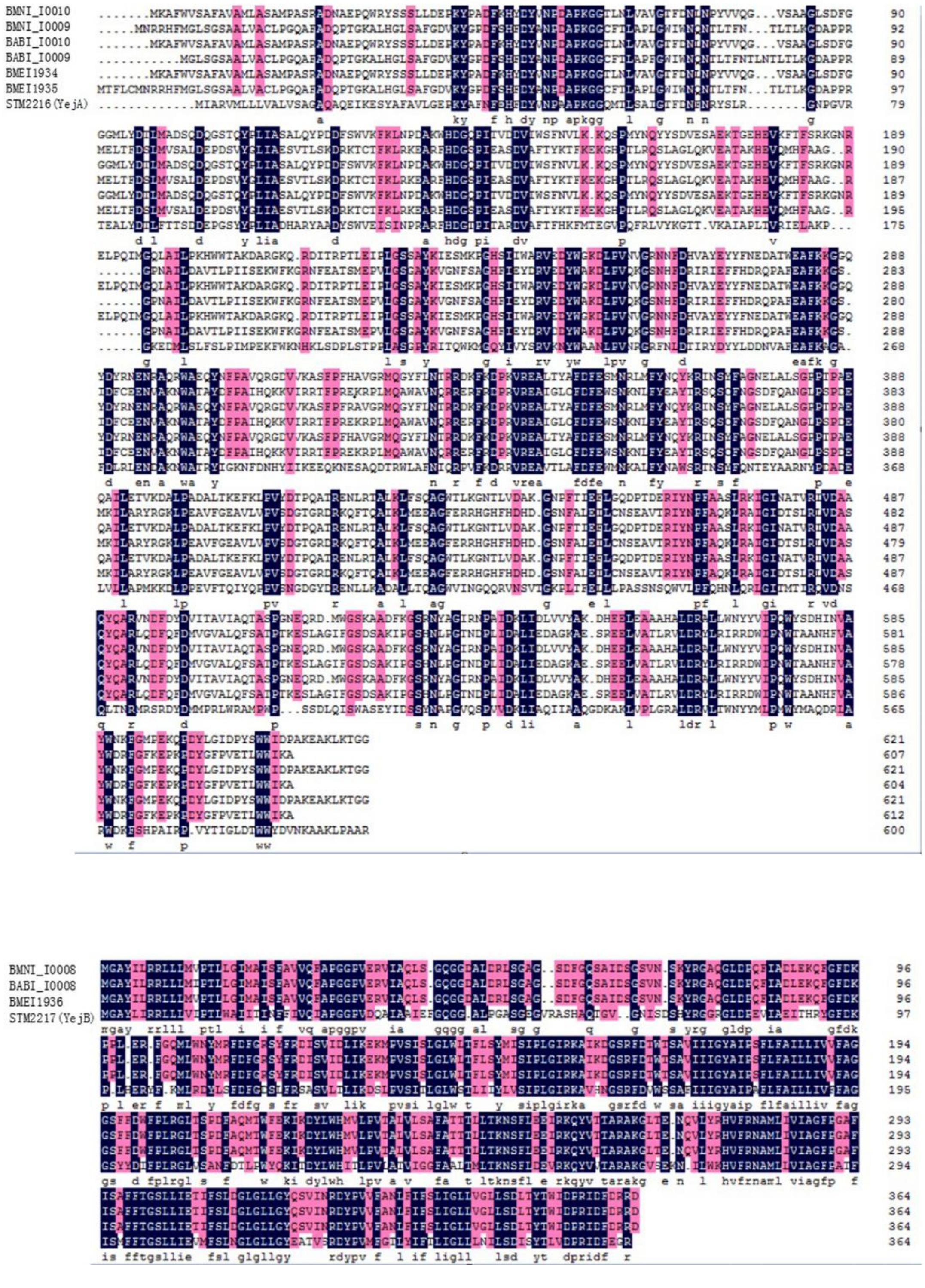
BMNI_I0010
BMNI_I0009
BABI_I0010
BABI_I0009
BMEI1934
BMEI1935
STM2216 (YejA)
BMNI_I0008
BABI_I0008
BMEI1936
STM2217 (YejB)

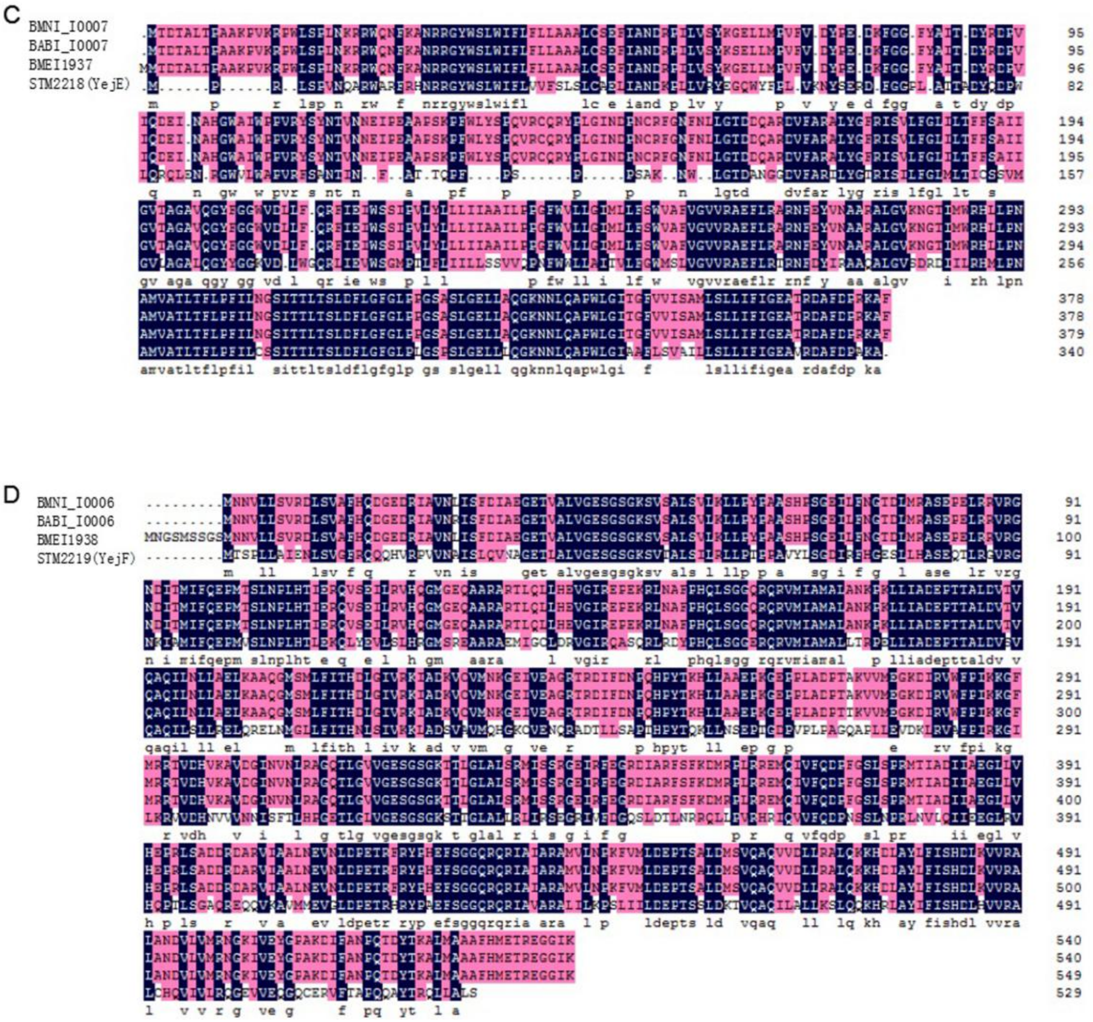


**Figure S1** Alignment of the amino acid sequences of Yej proteins of *Brucella* with them in *S. Typhimurium* LT2. (A) YejA, (B) YejB, (C) YejE, (D) YejF. Shadows of navy and pink indicated the amino acid sequence identities are 100%and 75%, respectively.The accession numbers of the *yej* operon genes in *B.melitensis* NI are, *yejA1*:GI:384444099, *yejA2*:GI:384444098, *yejB*:GI:384444097, *yejE*:GI:384444096, and *yejF*:GI:384444095, respectively; in *B.melitensis* 16M are, *yejA1*:GI:17988217, *yejA2*:GI:17988218, *yejB*:GI:17988219, *yejE*:GI:17988220, and *yejF*:GI:17988221, respectively; in *B. abortus* 2308 are, *yejA1*:GI:82698942, *yejA2*:GI:82698941, *yejB*:GI:82698940, *yejE*:GI:82698939, and *yejF*:GI:82698938, respectively; the accession numbers of the *yej* operon genes in *S. Typhimurium* LT2 are, *yejA*:GI:16765545, *yejB*:GI:16765546, *yejE*:GI:16765547, *yejF*:GI:16765548,respectively.
